# Follicular Atopic Dermatitis in Dark Skin

**DOI:** 10.7759/cureus.110093

**Published:** 2026-06-02

**Authors:** Salma Kozmane, Zakia Douhi, Sofia El Haitamy, Layla Tahiri Elousrouti, Fatima Zahra Mernissi

**Affiliations:** 1 Department of Dermatology, Faculty of Medicine, Pharmacy and Dental Medicine, Hassan II University Hospital, Sidi Mohamed Ben Abdellah University, Fez, MAR; 2 Department of Pathology, Hassan II University Hospital, Fez, MAR

**Keywords:** atopic dermatitis, dark skin, dermoscopy, follicular variant, pediatrics

## Abstract

Atopic dermatitis (AD) may present with distinct clinical features in individuals with dark skin, often posing diagnostic challenges. We report a pediatric case of follicular AD in a dark-skinned patient to highlight its characteristic clinical presentation. The eruption consisted of bilateral, symmetrical, multiple, discrete, purplish to brownish follicular scaly papules distributed over the trunk, back, and both upper and lower limbs. Dermoscopy revealed a white-brown background, normal-appearing terminal hairs in the center, perifollicular desquamation, multiple dotted vessels with heterogeneous distribution, and hemorrhagic crusts; histopathology confirmed the diagnosis. This report emphasizes the importance of recognizing these specific clinical and dermoscopic features of AD in dark skin to improve diagnostic accuracy and optimize management.

## Introduction

Atopic dermatitis (AD) is a widespread inflammatory skin disorder that occurs across various ethnic populations. Since the 1970s, the one-year prevalence of AD has risen two- to threefold in industrialized countries, now affecting roughly 15-20% of children, 5-20% of adolescents, and 1-3% of adults [1]. AD is traditionally characterized by clusters of pruritic papules and erythematous plaques, mainly affecting flexural areas and often accompanied by excoriations, oozing, and scaling in the subacute or chronic stages. However, the clinical appearance of AD may vary in people with dark skin, where erythema is often less apparent, and lesions may present as keratotic follicular papules [2]. Dermoscopy is a quick, non-invasive diagnostic tool that can aid in the diagnosis of AD and help differentiate it from conditions such as psoriasis, lichen planus, and Darier's disease. We report a rare case of follicular AD in a patient with a dark phototype, highlighting its diagnostic challenges and the correlation between dermoscopic and histopathological findings.

## Case presentation

**Figure 1:**
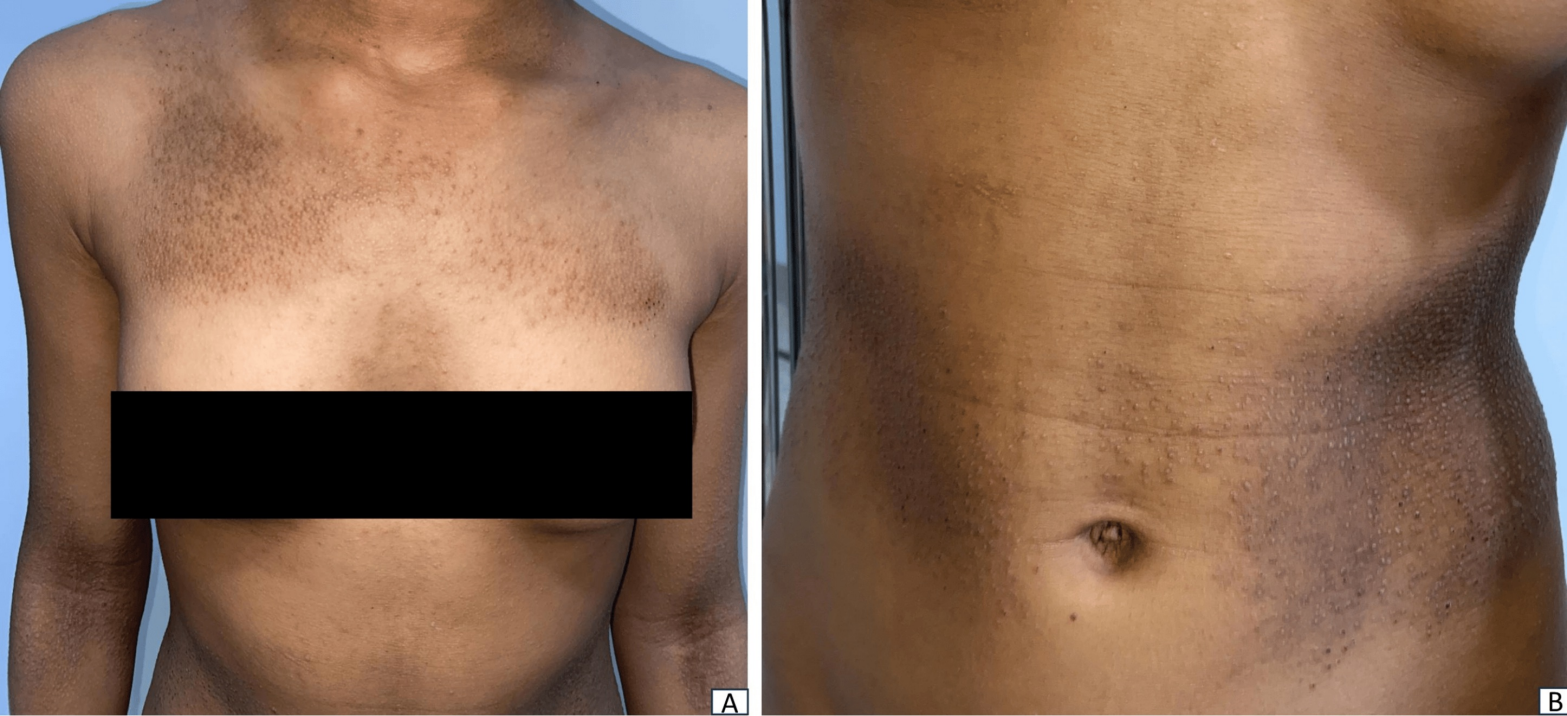
Patient frontal images (A) Multiple discrete, purplish to brownish follicular scaly papules with excoriated surfaces on the chest and elbow creases. (B) The same lesions on the abdomen

**Figure 2:**
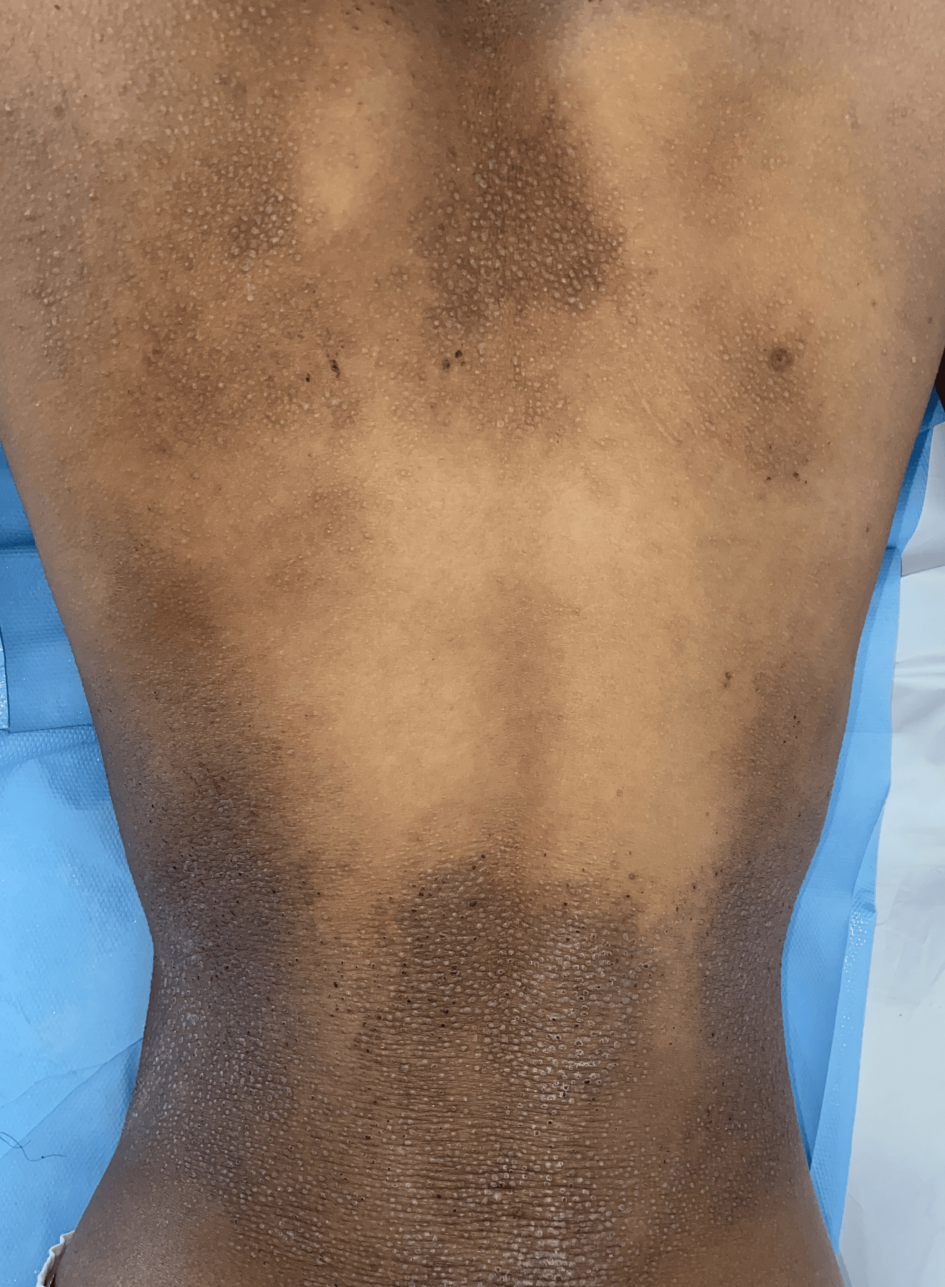
Multiple brown follicular papules on the back

A 14-year-old female presented with multiple pruritic, skin-colored to reddish, elevated lesions involving the trunk, back, and both the flexural and extensor aspects of the upper and lower limbs, which had been gradually developing for more than 10 years. There was a positive family history of allergic rhinitis. She denied any history of prior drug intake, scalp scaling, joint pain or swelling, or palmoplantar thickening. Cutaneous examination revealed skin phototype V with multiple discrete, purplish to brownish, follicular scaly papules distributed over the trunk, back, and both the upper and lower limbs (Figures 1A, 1B, 2).

**Figure 3:**
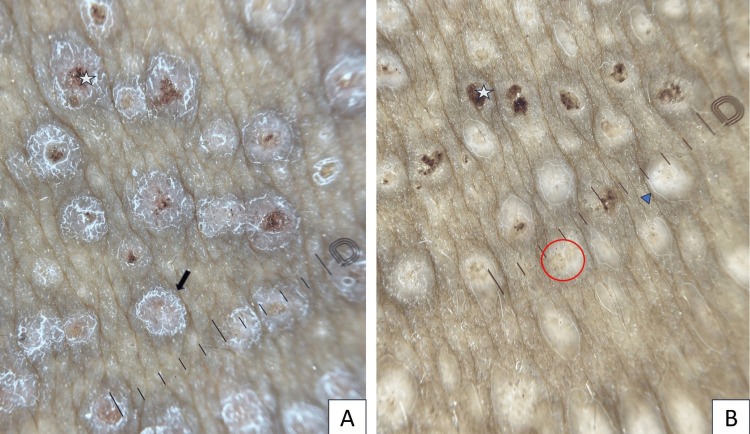
Dermoscopy images (A) Dermoscopy without immersion, showing peripheral scaling (black arrow) and hemorrhagic crusts (white star). (B) Dermoscopy with immersion, showing a white-to-brown background (blue triangle), hemorrhagic crusts (white star), and dotted vessels (red circle)

The mucosae, nails, scalp, palms, and soles were spared. Systemic examination was unremarkable. Differential diagnoses included follicular AD, follicular psoriasis, Darier's disease, and follicular lichen planus. Dermoscopic examination, performed using polarized dermoscopy with and without immersion (DermLite DL5 dermatoscope, DermLite, LLC, Aliso Viejo, CA), showed a white-brown background, normal-appearing terminal hairs in the center, perifollicular desquamation, multiple dotted vessels with heterogeneous distribution, and hemorrhagic crusts (Figures 3A, 3B).

**Figure 4:**
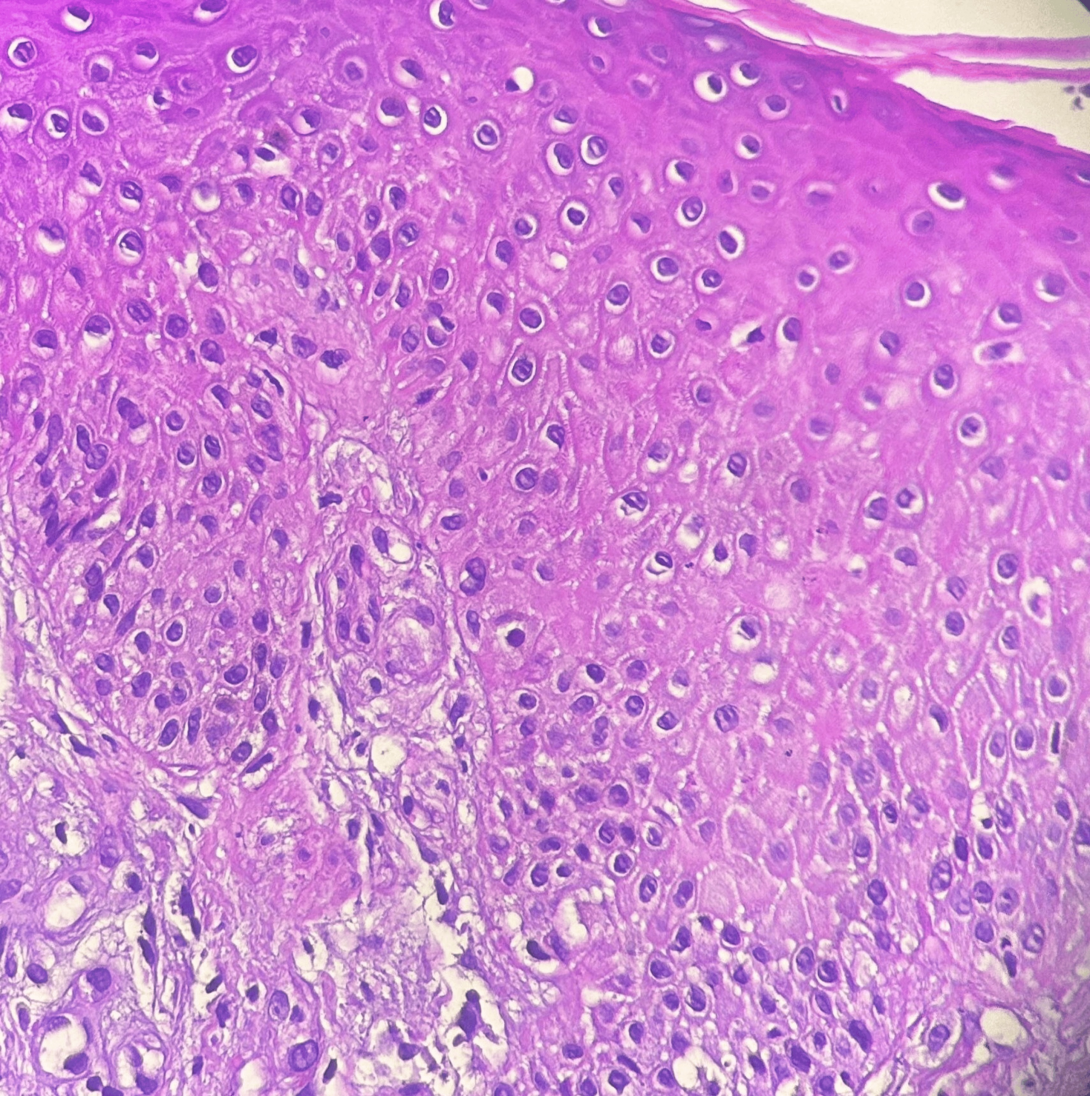
Histopathological findings Acanthotic and spongiotic epidermis covered by orthokeratotic scales, without hypergranulosis. The dermis showed a mild perivascular and interstitial inflammatory infiltrate, with no evidence of interface dermatitis (H&E stain, magnification x 400)

Histopathological examination of a papule revealed a slightly acanthotic and spongiotic epidermis, covered by orthokeratotic and occasionally parakeratotic scales, without hypergranulosis. The dermis showed a mild perivascular and interstitial inflammatory infiltrate, with no evidence of interface dermatitis (Figure 4).

Based on the patient’s history, particularly the positive family history of atopy, together with the clinical, dermoscopic, and histopathological findings, a diagnosis of follicular AD was established. The patient was treated with a topical combination of white petrolatum and betamethasone until the lesions resolved, followed by a proactive regimen including hygiene measures and regular moisturizing. Unfortunately, the patient was lost to follow-up.

## Discussion

AD is the most prevalent chronic inflammatory skin condition. Its prevalence varies greatly worldwide, ranging from 0.2% to 24.6% across different geographical regions. The International Study of Asthma and Allergies in Childhood (ISAAC) highlighted this variability, reporting higher prevalence rates in industrialized countries, particularly in Northern Europe and Oceania, and lower rates in regions such as Africa, India, and parts of Eastern Europe [3]. Both skin color and racial/ethnic background influence the clinical presentation of AD. Additionally, AD is a complex, multifactorial disorder that has recently been shown to exhibit unique phenotypic and molecular profiles across ethnic groups. According to one study, African American patients with AD have lower Th1 and Th17 responses, while their Th2 and Th22 pathways are upregulated similarly to those of European American patients [4].

Multiple phenotypes of AD have been identified based on the distribution of lesions according to age. In infants, lesions typically begin on the face and scalp and may extend to the extensor surfaces of the limbs and trunk, while the buttocks and nose are usually spared. After the age of two years, lesions tend to localize to the flexural folds of the limbs and often involve the ankles. In adolescents and adults, lesions are predominantly located on the face and neck and often become clearly lichenified on the limbs [5]. Regarding skin phototype, in individuals with fair skin, AD usually presents as erythematous lesions that are readily visible due to the contrast with surrounding skin, and the intensity of inflammation is generally easy to assess because the erythema is clearly apparent.

However, the clinical presentation can vary greatly in people with darker skin types (African, Asian, or Indian), which can occasionally result in a delayed or incorrect diagnosis. It's crucial to remember that while pruritus is a common symptom of all types of AD, patients with darker skin typically experience it more intensely than those with lighter skin [6]. In addition, follicular AD represents a distinct clinical variant, more frequently observed in patients with dark skin, and characterized by keratotic follicular papules with minimal visible erythema or even concealed by lesions that have a purplish, grayish, or dark brown appearance. Perifollicular accentuation and distinct papules distributed over the extensor surfaces and trunk are also more commonly observed in these individuals. As illustrated in our case, this follicular pattern may contribute to diagnostic difficulty due to its resemblance to other follicular dermatoses [6]. Other characteristic features in darker phototypes include dyspigmentation of the infraorbital folds, Dennie-Morgan lines, palmar hyperlinearity, and white dermographism [7].

The role of dermoscopy as a diagnostic tool is increasingly recognized, as an expanding number of dermatologic conditions are being identified in which dermoscopy contributes not only to diagnosis but also to monitoring disease progression and treatment response. Compared with fair-skinned individuals, AD in darker phototypes shows distinctive dermoscopic features related to increased pigmentation. While scales and dotted vessels remain common findings in both groups, darker phototypes more frequently exhibit brown pigmented structures, such as reticular lines, globules, clods, and brown dots. As reported in our case, the background is often brownish, pink rather than erythematous, and red. Erythema and vascular structures tend to be less apparent [8]. To the best of our knowledge, the dermoscopic features of follicular AD have been rarely reported in the literature. 

The follicular form of AD can be easily mistaken for lichen planus. In our patient, the notable differences from true lichen planus included a positive family history of atopy, round rather than polygonal papules, an absent Koebner phenomenon, absence of Wickham’s striae, and lack of mucosal, genital, or scalp involvement. The lesions were predominantly distributed over the extensor surfaces, and histopathological examination revealed eczematous rather than lichenoid changes [9].

Follicular psoriasis was also taken into consideration as a differential diagnosis. Erythematous, scaly follicular papules on the trunk and extremities are its defining feature. Sometimes accompanied by erythematous scaly plaques, the lesions can appear scattered or clustered. A perifollicular white homogeneous area, central terminal hairs that appear normal, perifollicular scaling, multiple dotted vessels, red globules, twisted red loops, and glomerular vessels are all visible on a dermoscopic examination of follicular psoriasis [10]. Under a microscope, it exhibits psoriasiform alterations that are limited to the hair follicles, such as acanthosis of the infundibular epithelium with loss of the granular layer and evident follicular plugging with parakeratotic scales containing neutrophils [11].

Finally, Darier's disease may also mimic follicular AD, as it presents with keratotic papules (rough, brownish) measuring 1-5 mm that may coalesce into verrucous plaques in seborrheic areas. However, our patient did not present with lesions in seborrheic regions and had no nail abnormalities such as red and white longitudinal striations, fragility, distal “V” shaped notching, or subungual hyperkeratosis, or mucosal lesions (white or yellowish papules on the oral or genital mucosa). Dermoscopy can assist in distinguishing Darier's disease by revealing a pinkish homogeneous background, central pseudocomedones, and vascular structures. In darker skin types, brown polygonal or round structures corresponding to hyperkeratosis and a surrounding whitish halo corresponding to acanthosis have been described as consistent findings [12-13].

## Conclusions

Our case illustrates follicular AD as an uncommon presentation in patients with darker skin phototypes, highlighting the diagnostic challenges posed by follicular variants in these individuals. The atypical clinical appearance, characterized by follicular keratotic papules with poorly visible erythema, may mimic several inflammatory disorders, including follicular psoriasis, lichen planus, or Darier's disease, potentially leading to misdiagnosis. Our report also emphasizes the complementary role of dermoscopy, which revealed a white-brown background, perifollicular desquamation, and dotted vessels with heterogeneous distribution. Furthermore, histopathological examination remains essential for improving diagnostic accuracy in challenging presentations.
